# Tolerance of ambiguity and psychological well‐being in medical training: A systematic review

**DOI:** 10.1111/medu.14031

**Published:** 2019-12-22

**Authors:** Jason Hancock, Karen Mattick

**Affiliations:** ^1^ Devon Partnership Trust Exeter UK; ^2^ University of Exeter Medical School, College of Medicine and Health University of Exeter Exeter UK

## Abstract

**Context:**

The prevalence of stress, burnout and mental health disorders in medical students and doctors is high. It has been proposed that there may be an association between levels of tolerance of ambiguity (ie an ability to tolerate a lack of reliable, credible or adequate information) in clinical work and psychological well‐being within this population. The aims of this systematic review were: (i) to assess the nature and extent of the literature available, in order to determine if there is an association, and (ii) to develop a conceptual model proposing possible mechanisms to underpin any association, in order to inform subsequent research.

**Methods:**

MEDLINE, Cumulative Index to Nursing and Allied Health Literature (CINAHL) and PsycINFO databases were searched for articles published from inception to September 2018. Additional literature was identified by searching the reference lists of included articles, forward searches of included articles, hand searches of key journals and a grey literature search. Of the 671 studies identified, 11 met the inclusion criteria. A qualitative synthesis of included studies was performed.

**Results:**

All 11 included studies reported an association between a lower level of tolerance of ambiguity or uncertainty and reduced psychological well‐being. Included studies were heterogeneous in terms of population and measurement approach, and were often of low methodological quality. Subsets of items from previously developed scales were often used without sufficient consideration of the impact of new combinations of items on scale validity. Similar scales were also scored inconsistently between studies, making comparison difficult.

**Conclusions:**

There appears to be an association between tolerance of ambiguity and psychological well‐being. This provides new opportunities to understand and prevent the development of stress, burnout and mental health disorders in medical students and doctors. The conceptual model developed provides a framework for future research, which we hope will prevent wasted research effort through duplication and promote higher methodological quality.

## Abstract 2

Although tolerance for uncertainty is broadly considered important for high quality care of patients, this review suggests it may also be associated with the wellbeing of doctors and students themselves.

## 1 INTRODUCTION

The prevalence of stress, burnout and mental health disorders, such as depression and anxiety, in doctors worldwide is alarmingly high.^1^, ^2^, ^3^, ^4^, ^5^, ^6^ Similarly, there is a high prevalence of depression and anxiety in medical students, and by the end of undergraduate training levels of ‘psychological distress’ are higher than in the age‐matched general population.^7^ This may lead to absenteeism (where doctors or students miss work or study due to their mental ill‐health), presenteeism (where doctors or students come to work or study when unwell) and loss of staff from the workforce (where doctors or students leave the profession of medicine altogether).^8^ Such implications have negative consequences for individual doctors or medical students, their current and future patients and their colleagues, and for the wider society, which may have shouldered much of the costs of their training.

Although the problems of stress, burnout and mental health disorders in doctors are well described, less is known about individual, team, organisation or societal factors that increase the risk of doctors developing these problems.^7^ It is likely that multiple factors contribute towards their increased prevalence in this population. A number of studies have proposed a link between intolerance of ambiguity or uncertainty in clinical practice and a range of outcomes, which could be considered under the broader term reduced psychological well‐being, including psychological distress, burnout and mental health disorders.^9^, ^10^, ^11^ Although it is widely acknowledged that ambiguity is inherent within the practice of medicine,^12^ a greater understanding of the implications of this has been slow to develop, partly due to the conceptual complexity.

Varying definitions of tolerance of ambiguity and uncertainty have been proposed and used to underpin measures of these constructs in medical undergraduate and qualified doctor populations. Recently, a conceptual model for understanding tolerance of ambiguity and uncertainty was proposed based on a review of 18 existing measures of tolerance of uncertainty and ambiguity.^13^ This review outlined some of the challenges with existing measures, such as their poor conceptual clarity (eg using the terms uncertainty and ambiguity interchangeably) or inconsistent use of definitions for these constructs. In response, Hillen et al set out their definition of tolerance of uncertainty as ‘the set of negative and positive psychological responses—cognitive, emotional and behavioral—provoked by the conscious awareness of ignorance about particular aspects of the world.’ They state that uncertainty is the response to either ambiguity, probability or complexity and define ambiguity as a ‘lack of reliable, credible or adequate information.’ We adopt these definitions for the current study as they precisely and explicitly distinguish between ambiguity and uncertainty, based on the current literature. They also set out a clear definition for what it means to ‘tolerate’ these two closely related constructs.^13^

We hypothesise that intolerance of ambiguity in medical students and doctors could place an individual at increased risk of experiencing reduced psychological well‐being. By this, we mean stress, burnout or a more persistent pathological state such as anxiety or depression, which are themselves considered to be a mental health disorder. Examining the broader concept of psychological well‐being would aid understanding of the role that these potentially more transient states (stress and burnout) could play in the development of a mental health disorder. However, the terms stress and burnout themselves are not without controversy. For example, although the term ‘burnout’ is commonly used there remains no universally accepted definition for this; thus, the validity of burnout scales is unclear.^14^

Given that the set and extent of pressures that doctors and medical students encounter may be unique to this population,^8^ it seems reasonable to examine this potential association within this population without extending our hypothesis to include other health care professionals at this stage. By synthesising the existing literature on this topic, we hope to draw conclusions about the potential association between tolerance of ambiguity and psychological well‐being and offer a conceptual model that can be tested through subsequent research. This is particularly important given that many of the included concepts appear to be inconsistently defined and overlapping. A conceptual model would help to advance the research field and save unproductive research effort. It would also take us closer to designing evidence‐based interventions that might support doctors in coping with their often inherently ambiguous medical work.

## 2 METHODS

The aims of this systematic review were: (a) to assess the nature and extent of the literature available, in order to determine if there is an association between levels of tolerance of ambiguity and psychological well‐being within medical students and doctors, and (b) to develop a conceptual model proposing possible mechanisms to underpin any association, in order to inform subsequent research. The *p*referred *r*eporting *i*tems for *s*ystematic *r*eviews and *m*eta‐*a*nalyses (PRISMA) framework has been used to help guide and ensure the high quality of this systematic review.^15^, ^16^

### 2.1 Information Sources and Search Strategy

The search protocol was developed by undertaking pilot database searches in MEDLINE and PubMed and discussions between the authors (JH and KM) and others (see Acknowledgements), to identify and refine search terms. We included search terms that would identify studies that evaluated levels of ‘stress’ or ‘burnout’, and studies that assessed evidence of a mental health disorder, based on the author's definitions of these terms. Given the challenges with existing measures of tolerance of ambiguity and uncertainty, and the evidence that these measures often overlap substantially,^13^, ^17^ we decided to include search terms that would identify studies evaluating levels of tolerance of ambiguity or uncertainty. It is important to note that although we include studies evaluating tolerance of both ambiguity and uncertainty in this review this does not mean that we are using these terms interchangeably, just that we are aware that terminology has not always been used precisely in the published literature. The search terms and strategy were then further refined and finalised through discussion with an information specialist (AB).

Searches of PsycINFO, Cumulative Index to Nursing and Allied Health Literature (CINAHL) and MEDLINE databases for articles published from inception to 3 September 2018 were conducted. These databases were selected to ensure that published mental health and qualitative literature were considered in addition to more traditional biomedical articles. Relevant Medical Subject Headings or subject terms were explored and included. No search limitations were applied at this stage. The full search strategies for each database are included in Table S1.

### 2.2 Study selection

**Figure 1:**
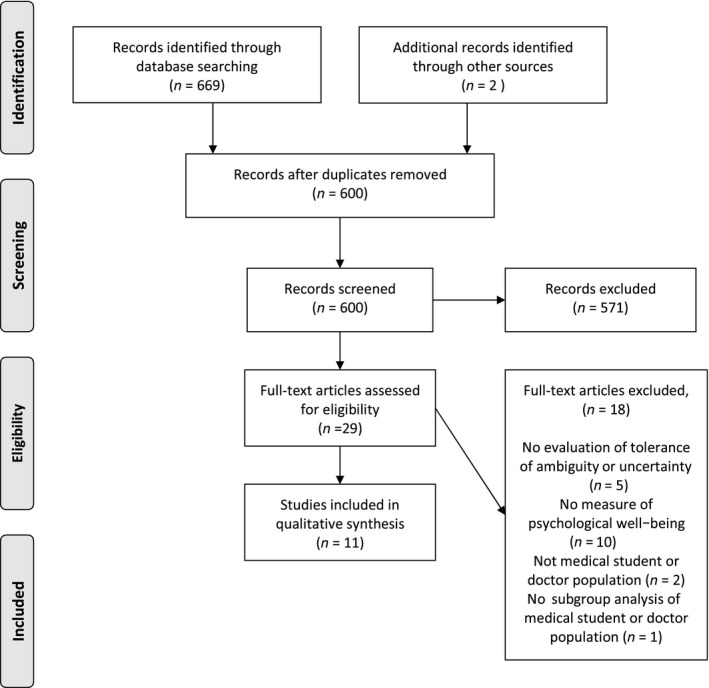
PRISMA (*p*referred *r*eporting *i*tems for *s*ystematic *r*eviews and *m*eta‐*a*nalyses) flow diagram

In total 669 papers were identified through the initial search of the three databases (see Figure 1). There were 71 duplicates, meaning that 598 papers were screened using the inclusion and exclusion criteria. Studies were included if they: (a) were an empirical study; (b) used any defined measure of ambiguity or uncertainty tolerance, (c) used a measure of psychological well‐being (stress, burnout or evidence of a mental health disorder); and (d) were conducted within the undergraduate medical student or postgraduate doctor population. These are defined in more detail in Table 1.

**Table 1:** Inclusion and exclusion criteria

Inclusion criteria	Exclusion criteria
Empirical study (peer‐reviewed article that presents quantitative and/or qualitative data)	Not published in English
Describes any quantitative or qualitative methodology used to evaluate tolerance of ambiguity or uncertainty	Contained medical students or doctors within the population but did not include a subgroup analysis that allowed these populations to be evaluated independently of other health care professionals
Describes methodology used to assess for evidence of psychological well‐being. This could include the presence of a mental health diagnosis (self‐rated or clinician assessed), any measure of psychological distress (self‐rated or clinician assessed), stress or burnout	
Conducted in either undergraduate medical students or postgraduate doctors	

JH and KM independently screened 75 (12.5%) of the titles and abstracts to determine if they met the inclusion criteria for further analysis or inclusion in the review. There was agreement on 74 out of 75 and in the one case where there was disagreement this was resolved through discussion. The remaining 523 papers were screened by JH alone, with further discussion with KM where needed.

Of the 598 papers that were screened, 571 were excluded following a review of the title and abstract as they did not meet the inclusion criteria for the review. Full papers were requested and reviewed for 27 studies. Of these 27 full papers, 17 did not meet the inclusion criteria, meaning that 10 papers in total were included.

### 2.3 Supplementary and grey literature searches

Reference lists from each of these 10 studies were examined for potential additional papers. JH also performed a ‘hand search’ of article titles and abstracts for all papers published online or in print between 1 January 2018 and 24 October 2018 for each of the journals contributing a paper to the review. In addition, ‘forward searches’ were performed for each of the 10 papers included in the review by JH using Google Scholar (9 January 2019) and additional searches were conducted using Google Scholar. Finally, websites of professional regulators, professional bodies and relevant societies were searched for published or commissioned reports; see Table S2 for full details of all supplementary and grey literature searches.

Through this process, two additional studies were identified. One was excluded because, although physicians were included in the study population, it was not possible to evaluate their scores independently of other health care professionals. One study met the inclusion criteria and was included in our final review.

### 2.4 Data extraction and summary of findings

Relevant information, including country, sample, study design, measure of tolerance of ambiguity or uncertainty and measure of psychological well‐being and outcomes, was extracted from each included article by JH.

### 2.5 Quality assessment

Study quality was appraised using the Medical Education Research Study Quality Instrument (MERSQI).^18^ The MERSQI evaluates study quality based on study design, sampling, type of data, validity of evaluation instrument, data analysis and outcomes. Each item is scored on a scale of 1–3 and summed to determine a total score. The maximum score for each domain was 3, therefore the maximum MERSQI score is 18 with a potential range of 5–18. The total MERSQI score was calculated as the percentage of total achievable points (accounting for ‘non‐applicable’ responses) and then adjusted to a standard denominator of 18 to allow for comparison of scores across studies.

## 3 RESULTS

The 11 included studies, along with the quality assessment score, are summarised in Table 2. Of these, 10 were cross‐sectional and one was longitudinal. Studies were heterogeneous in terms of professional populations and country of study. Four studies were conducted with medical students, one at matriculation into medical school, one in the fourth year, one across all years and one at graduation. The remaining studies involved general practitioners (GPs) or primary care physicians (3), emergency physicians (2), paediatricians (1) and physicians (1). Studies were conducted in the USA alone (5), North America (1), Ireland (1), Italy (1), Australia (1), Finland (1) and Switzerland (1). The 11 studies involved a total of 15 353 participants (sample sizes from 47 to 13 314). The largest study (*n* = 13 314) was conducted on matriculating medical students in medical schools across the USA. One study of USA medical students contained 739 participants. The other nine studies had 212 participants or fewer.

**Table 2:** Summary of included studies

Author (year); country	Title	Population (*n*)	Study design	Measure of tolerance of uncertainty/ambiguity	Measure of psychological well‐being	Main findings	Study quality. MERSQI score out of 18
Bachman et al (1999); USA^22^	HMO physicians’ use of referrals	Primary care physicians (paediatrics, family practice or general internal medicine) working within two health maintenance organisations in the USA (212)	Cross‐sectional, multicentre	*Uncertainty*: Physicians’ Reactions to Uncertainty (PRU) Scale (1990)^11^ score calculated	*Burnout*: Tedium Index^38^ Score calculated	Stress from uncertainty and reluctance to disclose uncertainty both correlated with burnout: *SUS: r = .38,* p* = .0001*;* RDUS: r = .31,* p* = .0001* Using multivariate methods, stress from uncertainty (p *= .0001*) and reluctance to disclose uncertainty (p* = .0518*) remained predictors of burnout.	9
Caulfield et al (2014); USA^30^	Ambiguity tolerance of students matriculating to USA medical schools	Matriculating medical students across all USA medical schools (13314)	Cross‐sectional, multicentre	*Ambiguity*: Tolerance for ambiguity (TFA)^29^ score calculated	*Stress*: Perceived Stress Scale (PSS)^43^ 10 items self‐reported stress over last month Placed into quartiles based on score	Those expressing higher levels of stress reported lower tolerance for ambiguity Difference in average TFA score between first quartile PSS score (0‐8) and fourth quartile group (15‐40) showed a medium effect size: *27.1 (5.8) vs 23.3 (5.5) (d = 0.66)*	10.2
Cooke et al (2013); Australia^24^	A survey of resilience, burnout and tolerance of uncertainty in Australian general practice registrars	General practitioner (GP) registrars in training across four regional training providers (128)	Cross‐sectional, multicentre	*Uncertainty*: Intolerance of Uncertainty‐12 (IUS‐12)^28^ score calculated PRU Scale (1995)^23^ score calculated	*Burnout*: Single‐item measure of burnout validated against the Maslach Burnout Inventory (MBI)^36^ High risk of burnout if elected statements 3–5 Professional Quality of Life (ProQOL)^37^ scale measures burnout, secondary traumatic stress, compassion satisfaction	GP registrars deemed to be at high risk of burnout had higher intolerance of uncertainty compared to those deemed at low risk of burnout (IUS‐12): *36.6 (9.8) vs 30.2 (7.2)* p* = .001* GP registrars deemed to be at high risk of burnout had higher levels of anxiety due to uncertainty: *21.7 (4.3) vs 18.8 (5.0)* p* = .02,* and greater reluctance to disclose uncertainty to patients: *15.7 (3.3) vs 13.6 (3.8),* p *= .03,* compared to those deemed at low risk of burnout (PRU) No significant difference in ‘concern about bad outcomes’ or ‘reluctance to disclose uncertainty to physicians’ in those deemed at high risk of burnout compared to those at low risk	9.6
Iannello et al (2017); Italy^19^	Ambiguity and uncertainty tolerance, need for cognition and their association with stress. A study amongst Italian practising physicians	Practising physicians of various specialties and experience (212) across 11 hospital sites	Cross‐sectional, multicentre	*Ambiguity*: TFA^29^ score calculated *Uncertainty*: PRU Scale – ‘Stress from uncertainty’ component (1990)^11^ score calculated	*Perceived job stress*: Job Stress Questionnaire (JSQ)^42^ Score calculated	Work‐related stress had a ‘moderate negative correlation’ with tolerance of ambiguity Work‐related stress had a ‘moderate positive correlation’ with level of stress for uncertainty Hierarchical linear regression model ‘stress from uncertainty’ strongest predictor of work‐related stress	9.6
Klem et al (2014); Switzerland^34^	Positive interpretation bias predicts well‐being in medical interns	Final‐year medical students about to start as medical interns (47)	Longitudinal (6 months), single centre	*Ambiguity*: Ambiguous Scenario Task AST‐D at baseline^35^ Participants considered to have positive interpretation bias (scored above midpoint of scale) or negative interpretation bias (scored below midpoint of scale)	*Depression*: Patient Health Questionnaire (PHQ‐9)^41^ used to assess symptoms of depression at 0, 3, 6 months. Participants with score ≥ 5 considered to have depression	Baseline depressive symptoms at T0 significantly associated with negative interpretation bias (*R* ^*2*^ *= 0.23,* p *< .001*) Tendency to interpret ambiguous scenarios in a more positive manner was associated with a sixfold decreased risk of depressive symptomology (*odds ratio [OR] 6.25, 1.2‐33.3*) and associated with heightened depressive symptoms severity at 6 months (β* = −0.34,* p* = .027*); this remains the case even when controlling for initial depression and trait reappraisal	9
Kuhn et al (2009); USA^25^	Tolerance of uncertainty, burnout and satisfaction with the career of emergency medicine	Emergency physicians registered with American College of Emergency Physicians (ACEP) (193)	Cross‐sectional, multicentre	*Uncertainty*: PRU Scale (1995)^23^ Participants classed as having ‘discomfort with uncertainty’ for each subscale if participant scored 75th percentile or above	*Burnout*: MBI^36^ Participants classed as ‘high’ burnout if scored high on any component of scale	Those with high burnout more likely to exhibit anxiety caused by uncertainty (*unadjusted OR 3.3*) and concern about bad outcomes (*unadjusted OR 7.7*) When multivariable logistic regression model constructed using all variables related to workplace dissatisfaction and discomfort from uncertainty, high anxiety caused by concern over bad outcomes (*OR = 6.35*) single greatest predictor of career burnout	8.4
Lally et al (2014); Ireland^20^	Uncertainty and ambiguity and their association with psychological distress in medical students	Fourth‐year medical students at one medical school in Ireland (100)	Cross‐sectional, single site	*Ambiguity:* Modified version of Tolerance for Ambiguity (Budner)^31^ Four items reworded for medical context; score calculated *Uncertainty:* Intolerance of Uncertainty Scale (IUS‐12) (Carleton et al)^28^ score calculated	*Psychological distress*: GHQ‐12 – General Health Questionnaire 12^44^ Score > 3 considered a case of psychological distress	Students with psychological distress had a higher mean intolerance of uncertainty score compared with those without psychological distress: *31.7 (6.18) vs 26.66,* p* < .001* Students with psychological distress had no statistically significant difference in their tolerance of ambiguity score compared with those without psychological distress*: 42.74 (6.55) vs 40.37 (6.67)* p* = .116*	10.2
Mangione et al (2018); USA^33^	Medical students’ exposure to the humanities correlates with positive personal qualities and reduced burnout: a multi‐institutional USA Survey	All medical students enrolled in five USA medical schools (739)	Cross‐sectional, multicentre	*Ambiguity*: Full version of Tolerance for Ambiguity Scale (Budner)^31^ score calculated	*Burnout*: Three subscales of the Shirom‐Melamed Burnout Measure: physical fatigue, cognitive weariness and emotional exhaustion^39^ scores calculated	Negative correlation between tolerance for ambiguity and emotional exhaustion *B* = −0.14 *(*p* < .01)* No statistically significant relationship identified between tolerance for ambiguity and cognitive weariness, *B* = 0.01, and physical fatigue, *B* = −0.05	8.4
Simpkin et al (2018); North America (USA and Canada)^26^	Stress from uncertainty and resilience amongst depressed and burned‐out residents: a cross‐sectional study	Paediatric residents (50) across four hospital sites	Cross‐sectional, multicentre	*Uncertainty*: PRU Scale (1995)^23^ ‘Stress from uncertainty’ calculated from three components: anxiety caused by uncertainty; concern about bad outcomes, and reluctance to disclose uncertainty to patients	*Depression*: Harvard National Depression Screening Day Scale^40^ Considered depressed if score ≥ 9 *Burnout*: MBI^36^ Two components used: emotional exhaustion and depersonalisation Considered burned out if experience either item at least weekly	Depressed residents more likely to have increased ‘stress from uncertainty’ than non‐depressed residents *(51.6 (9.07) vs 38.7 (6.7),* p* < .001)* Burned‐out residents significantly more likely to have increased stress from uncertainty than non‐burned‐out residents *(44 (8.46) vs 38.3 (7.1),* p *= .02)*	9
Takayesu et al (2014); USA^27^	Factors associated with burnout during emergency medicine residency	Emergency medicine residents across eight training programmes (193)	Cross‐sectional, multicentre	*Uncertainty*: PRU Scale (1995)^23^ score calculated.	*Burnout*: MBI^36^ Considered ‘high burnout’ if high emotional exhaustion, high depersonalisation, or low personal accomplishment	Intolerance of uncertainty correlated significantly with burnout Low burnout uncertainty score *42.3 (9.9)* vs high burnout uncertainty score *45.8 (9.9)*, p* = .015*	9
Torppa et al (2015); Finland^21^	Emotionally exhausting factors in general practitioners’ work	GPs from junior doctor to specialist doctor (165) across multiple sites	Cross‐sectional, multicentre	*Uncertainty*: Single‐item self‐reported questionnaire. ‘How do you tolerate uncertainty when making medical decisions’; three‐item response: ‘well’, ‘quite well’, ‘poorly’	*Emotional exhaustion*: MBI^36^: ‘I feel burnt out from my job’ Considered emotionally exhausted if answered ‘quite often’ or ‘often’	Larger proportion of emotionally exhausted GPs than of non‐emotionally exhausted GPs tolerated uncertainty poorly (*10% vs 2%*, p *= .040*) Logistic regression analysis showed that tolerance of uncertainty protected against emotional exhaustion (*OR 0.2, 95% CI 0.09‐0.7,* p* = .010*)	9

In all 11 included studies there was a reported association between a higher level of intolerance of ambiguity or uncertainty and reduced psychological well‐being. We now present results for the measurement tools used for tolerance of ambiguity or uncertainty, the measurement tools used to evaluate psychological well‐being and the associations identified between tolerance of ambiguity and measures of psychological well‐being.

### 3.1 Tolerance of ambiguity or uncertainty

A number of measurement approaches were used to assess levels of tolerance of ambiguity or uncertainty in the included studies (Table S3). Tolerance of uncertainty was assessed in eight studies and tolerance of ambiguity was assessed in five studies. Two studies assessed levels of tolerance of both ambiguity and uncertainty.^19^, ^20^

In 10/11 studies, previously validated scales (either the complete scales or a selected component) were used and in the remaining study a new ad hoc single‐item self‐reported questionnaire was used.^21^ The Physicians’ Reactions to Uncertainty (PRU) Scale, which evaluates physicians’ ‘affective’ response to uncertainty, was most frequently used (six studies). The 1990 version of this scale^11^ was used in two studies^19^, ^22^ and the 1995 version^23^ was used in four studies.^24^, ^25^, ^26^, ^27^ The Intolerance of Uncertainty Scale (IUS‐12)^28^ was used in two studies.^20^, ^24^ The Tolerance for Ambiguity Scale (Geller)^29^ was used in two studies.^19^, ^30^ The original Tolerance for Ambiguity Scale (Budner)^31^, ^32^ was used in one study,^33^ whereas a modified version of this scale was used in another study.^20^ The Ambiguous Scenario Task (AST‐D) was used in one study.^34^, ^35^ This differed from the other scales used as it evaluated an individual's interpretation bias (dichotomised into either positive or negative) in response to ambiguity.

In eight of the studies, level of tolerance of ambiguity or uncertainty was treated as a continuous variable, with a ‘score’ for tolerance of ambiguity or uncertainty being calculated based on scale responses. In three of the studies, level of tolerance of ambiguity or uncertainty was treated as an ordinal variable.^21^, ^25^, ^34^ In one of these studies^34^ scores above the midpoint were considered to represent a positive interpretation bias towards ambiguous situations, whereas in another^25^ participants were only considered to be tolerant of uncertainty if participants scored above the 75th centile.

### 3.2 Evaluation of psychological well‐being

Psychological well‐being was assessed using a range of different measurement approaches (Table S4). Self‐reported burnout was assessed in seven of the studies. Variations of the Maslach Burnout Inventory^36^ were used in five of the seven studies.^21^, ^24^, ^25^, ^26^, ^27^ This is a self‐reported measure of burnout that defines burnout as emotional exhaustion, depersonalisation and reduced personal accomplishment. Although this measure was used in five studies, a range of variations or components of the scale were used. In addition, interpretation of the scale to determine if an individual was ‘burned out’ was not consistent across studies. In one of these studies,^24^ the Professional Quality of Life (ProQOL) tool^37^ was also used as a measure of burnout. In one study, the Tedium Index^38^ was used to assess level of self‐reported burnout.^22^ In another,^33^ the Shirom‐Melamed Burnout Measure was used.^39^

Evidence of depression was evaluated in two studies: the Harvard National Depression Screening Day Scale^40^ was used in one study,^26^ and the PHQ‐9^41^ in one study.^34^ Self‐reported stress was assessed in two studies, with the ‘Job Stress Questionnaire (JSQ)’^42^ being used in one study^19^ and the ‘Perceived Stress Scale’^43^ being used in the other study.^30^ The General Health Questionniare‐12,^44^ a measure of psychiatric morbidity, was used in one study.^20^

### 3.3 Associations between tolerance of ambiguity and psychological well‐being

In all 11 included studies there was a reported association between a lower level of tolerance of ambiguity or uncertainty and reduced psychological well‐being.

In all studies where self‐reported burnout was assessed^21^, ^22^, ^24^, ^25^, ^26^, ^27^, ^33^ an association was demonstrated with lower levels of tolerance of ambiguity or uncertainty. Only one study measuring burnout evaluated levels of tolerance of ambiguity.^33^ The 1995 version of the PRU Scale^23^ claims to measure several different emotional or behavioural responses to uncertainty, including ‘anxiety caused by uncertainty,’ ‘concern about bad outcomes,’ ‘reluctance to disclose uncertainty to patients’ and ‘reluctance to disclose mistakes to physicians.’ Different studies demonstrated different relationships between the type of intolerance of uncertainty and self‐reported burnout. One study (*n* = 193) showed that for emergency physicians in the USA the ‘anxiety due to uncertainty’ and ‘concerns about bad outcomes’ components of the PRU scale appeared to be linked with burnout, but not ‘reluctance to disclose uncertainty’ or ‘reluctance to disclose mistakes to physicians.’^25^ Another study (*n* = 128) showed that for GP registrars in Australia ‘anxiety due to uncertainty’ and ‘reluctance to disclose uncertainty to patients’ appeared linked to burnout, but not ‘concern about bad outcomes’ or ‘reluctance to disclose mistakes to physicians.’^24^

Two studies attempted to identify if participants had evidence of depression. One showed that depressed paediatric residents in North America were more likely to have increased ‘stress from uncertainty’ than residents without depression.^26^ In this study, depression was defined as a score of ≥ 9 on the Harvard National Depression Screening Day Scale^40^ (51.6 [9.07] vs 38.7 [6.7], *P *<* *.001). Another study found that a tendency to interpret ambiguous scenarios in a more positive manner was associated with a sixfold decreased risk of experiencing depressive symptomology at 6 months.^34^ In this study evidence of depression was identified using the PHQ‐9^41^ (OR [odds ratio], 6.25; 1.2–33.3). This was the only study that was longitudinal in design.

One study showed that in Italian physicians self‐reported work‐related stress had a ‘moderate negative correlation’ with tolerance of ambiguity^19^ (using the Geller Scale of Tolerance for Ambiguity)^29^ and a ‘moderate positive correlation’ with level of stress for uncertainty (using the ‘stress from uncertainty’ component of the PRU 1990 scale).^11^ One study showed that in matriculating medical students those expressing higher levels of stress over the past month on the Perceived Stress Scale^43^ reported lower tolerance of ambiguity on Geller's Tolerance for Ambiguity Scale.^29^, ^30^ This was the largest study identified within this review (*n* = 13 314).

One study showed that students with psychological distress, evidenced by a General Health Questionnaire (GHQ‐12)^44^ score > 3, had a higher mean intolerance of uncertainty score compared with those without psychological distress using the Intolerance of Uncertainty Scale (IUS‐12): 31.7 vs 26.66, *P* < .001.^20^, ^28^ However, there was no difference in their tolerance of ambiguity scores when a modified version of the Tolerance for Ambiguity (Budner) Scale was used. This modified version of the Budner scale involved four of the original 16 scale items being used. The wording for each of these four items was also changed, as they had been reworded for a medical context.

### 3.4 Additional variables

In addition to evaluating tolerance of ambiguity and levels of psychological well‐being, additional variables were assessed in a number of the included studies. ‘Resilience’ was assessed in three studies.^24^, ^26^, ^34^ In two of these studies^24^, ^26^ those identified as being ‘burned out’ had lower self‐reported resilience and doctors with higher tolerance of uncertainty had higher self‐reported resilience scores. Both of these studies used the Resilience Scale‐14. This measures global resilience through evaluating five individual characteristics: (i) purpose; (ii) perseverance; (iii) self‐reliance; (iv) equanimity, and (v) existential aloneness.^45^ In one of these studies^26^ doctors with depression, identified on the Harvard National Depression Screening Day Scale,^40^ were found to have lower resilience scores. The final study showed that trait resilience was associated with positive interpretation bias in ambiguous situations (itself associated with a reduced risk of depression). This study was the only study that was longitudinal in design with interpretation bias in response to ambiguity and resilience being measured at baseline and evidence of depression being evaluated at 6 months.^26^

One paper^27^ looked at other factors that may be associated with burnout within emergency medicine residents in the USA. In addition to describing a significant correlation between intolerance of uncertainty and burnout, this paper also identified that those residents with a significant other or spouse had a higher prevalence of burnout compared to single residents (60% vs 40%, *P *=* *.002), and that other features such as lack of administrative autonomy and lack of clinical autonomy were also correlated with risk of burnout. Another paper^21^ reported that ‘feeling alone at work’ was associated with burnout (emotional exhaustion). However, in both of these papers these additional variables were not found to be associated with levels of tolerance of ambiguity or uncertainty.

### 3.5 Quality of studies

The overall methodological quality of studies was low. Total adjusted MERSQI scores amongst the 11 studies ranged from 8.4 to 10.2, with a mean (standard deviation) of 9.2 (0.62). All studies were observational and used self‐reported questionnaires to assess outcomes, with only one of the included studies being longitudinal in design.

## 4 DISCUSSION

The aims of this systematic review were: (i) to assess the nature and extent of the literature available, in order to determine if there is an association between levels of tolerance of ambiguity and psychological well‐being within medical students and doctors, and (b) to develop a conceptual model proposing possible mechanisms to underpin any association, in order to inform subsequent research. Although individual studies have demonstrated an association between intolerance of either ambiguity or uncertainty and stress, burnout or mental health disorders, this is the first systematic review of this topic and the first to synthesise the existing literature under the broader concept of psychological well‐being. The key finding was that the included studies appear to suggest an association between intolerance of ambiguity and reduced psychological well‐being.

Given the study designs involved, the heterogeneity of measurement approaches used and the different populations studied, it is not possible to draw firm conclusions about the direction of causality or strength of association. However, this was not the intention of this systematic review. Three different self‐reported scales were used in the five studies that measured tolerance of ambiguity, despite a recent study questioning the appropriateness of two of these scales within these populations.^46^ Three different self‐reported scales were used in the eight studies that measured tolerance of uncertainty, typically using components or subsets of previously validated scales, and novel and inconsistent approaches to scale interpretation. Four different versions of the PRU Scale were used across six studies. The Maslach Burnout Inventory (MBI) was the main measure used to assess burnout but, again, this was scored inconsistently. Unfortunately, there appeared to be little justification regarding changes to scale design, item inclusion, scoring or consideration of the impact this may have on scale validity in the population studied. This is problematic as it has been demonstrated that seemingly small technical decisions in scale design in this field can have significant implications for the findings.^47^

### 4.1 Conceptual model

**Figure 2:**
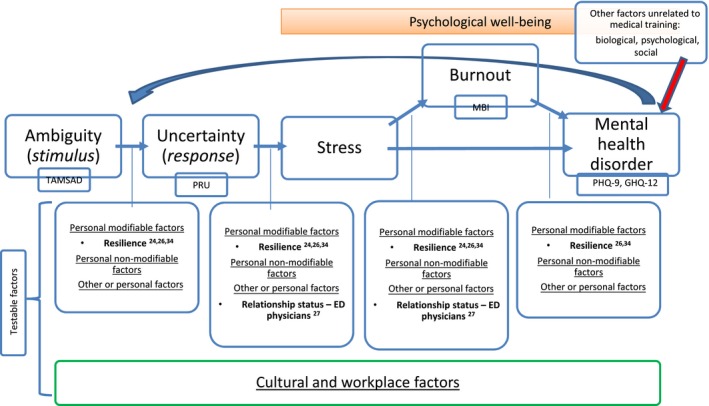
Conceptual model linking ambiguity tolerance to psychological well‐being. Abbreviations: ED, emergency department; GHQ‐12, General Health Questionnaire‐12; MBI, Maslach Burnout Inventory; PHQ‐9, Patient Health Questionnaire‐9; PRU, Physicians' Reactions to Uncertainty Scale; TAMSAD, Tolerance of Ambiguity in Medical Students and Doctors Scale [Colour figure can be viewed at http://www.wileyonlinelibrary.com]

Like other authors,^10^ we believe more carefully conceptualised and rigorously designed research programmes are now needed to progress this important area of research. We have therefore developed a conceptual model (Figure 2) based on our findings, which we offer as a starting point for future research. Thus, future studies might explore the direction and strength of the relationships highlighted by the model, between tolerance of ambiguity, tolerance of uncertainty and psychological well‐being, and the personal and wider workplace factors that may influence the individual outcomes.^8^

The model builds on work by Hillen et al,^13^ and incorporates the included studies from this systematic review and our a priori knowledge as researchers (KM and JH) and a consultant psychiatrist (JH). Ambiguity is defined as one of the potential causes of uncertainty.^13^, ^46^ Our systematic review found that there appears to be an association between intolerance of ambiguity and uncertainty and reduced psychological well‐being (stress, burnout or a mental health disorder) in medical students and doctors. This is indicated in the model with unidirectional arrows, reflecting our hypothesis that intolerance of ambiguity could be a factor in the development of reduced psychological well‐being, which needs testing through further research.

Stress was defined differently across studies^19^, ^30^ and its definition is more wide ranging than the more consistently defined pathological states of depression and anxiety. Subsequently, its position in the model is tentative. As has already been discussed there remains debate regarding the term ‘burnout’ and if this should also be considered a pathological state in itself. The most consistently used measure of burnout across the studies^21^, ^24^, ^25^, ^26^, ^27^ was the Maslach Burnout Inventory,^36^ which considers burnout to include features of emotional exhaustion, depersonalisation and reduced personal accomplishment. It is possible to have these features and not meet criteria for a mental disorder such as depression or anxiety. Equally it is possible to fulfil the criteria for both burnout and depression or anxiety. We hypothesise that some medical students and doctors may develop burnout prior to developing a mental health disorder; however, others may develop a mental health disorder without previously experiencing workplace‐associated burnout. Medical students and doctors may move through a range of psychological responses from an inability to tolerate ambiguity to intolerance of uncertainty, stress, burnout or the development of a mental health disorder. However, they may not progress through each stage and may not progress at all.

The likelihood of an individual progressing along this pathway, along with the factors that may increase or reduce this risk, requires future research. We have termed these factors as modifiable^24^, ^26^ (eg resilience), non‐modifiable (eg gender and age) or personal (eg relationship status).^27^ Until recently the organisation and structural contexts (eg workplace and cultural factors) that impact on a doctor's well‐being have been neglected. Therefore, the potential role that these may play in the development of reduced psychological well‐being is included in the model and is an important potential area for future research.^8^ The already established biological, psychological and social factors that influence the likelihood of developing a common mental health condition such as depression or anxiety are also reflected, but are beyond the scope of this model.

The challenges and issues associated with the measurement tools used in the established literature have already been discussed. This model includes recommendations for the measurement tools that could be used in future studies in order to promote more consistent higher quality research. This includes the use of the ‘Tolerance of Ambiguity in Medical Students and Doctors Scale’ (TAMSAD), which evaluates level of tolerance of ambiguity in medical students and postgraduate trainees,^46^ and the PRU Scale, which evaluates a physicians’ ‘affective’ response to uncertainty.^11^ We have also highlighted some established measures of mental health disorders, such as the PHQ‐9 (for depression) and the GHQ‐12 (to identify common psychiatric conditions) and suggest that they may be included in future studies.

### 4.2 Strengths and limitations of the study

The strengths of this review are its exhaustive search of the peer‐reviewed and grey literature, the careful synthesis of a complex and diverse literature into a clear picture that can inform future research and policy, and the development of a conceptual model that can promote high‐quality future research and potentially avoid wasted research effort.

We decided to include all studies that used any defined measure of tolerance of ambiguity or uncertainty, or of psychological well‐being. This ensured that we captured a wide range of published research but does mean that the included studies may be evaluating slightly different constructs, depending on the definitions adopted and the scales used. The interchangeable and inconsistent use of the terms uncertainty and ambiguity in the existing research has already been discussed.^13^ The concept of burnout also differs between countries, cultures and studies. For example, in some countries it can be regarded as a medical diagnosis, whereas in other countries it is used as a non‐medical label that carries minimum stigma.^48^ The self‐reported nature of the scales used presents further challenges in terms of the risk of over or under‐reporting psychological well‐being, and the impact that suffering from an issue such as stress, burnout or mental health disorder may have on the likelihood of agreeing to participate in the individual studies.

In addition to the varied definitions adopted by the included studies and the self‐reported nature of many of the scales used, our findings are also limited by the quality of the published research (eg small sample size and cross‐sectional design). The MERSQI tool was selected for its ability to evaluate the study designs in medical education; however, we still experienced significant challenges using this tool. For example, we stated ‘not applicable’ for the validity of evaluation instrument component because due to limited reporting it was difficult to compare the quality of the measurement approaches between studies. In addition, there is a significant risk that this review may have been influenced by publication bias, as studies that failed to identify an association between tolerance of ambiguity or uncertainty and mental health morbidity may have been less likely to be published.

### 4.3 Implications for policy and practice

Further research is required before the implications for policy and practice can be discussed with confidence. It does seem likely, however, that workplace cultures and environments might be designed in such a way that would reduce the likelihood that an individual's intolerance of ambiguity progresses to problems with their psychological well‐being. This could include modification of undergraduate or postgraduate medical training programmes, including the delivery of clinical supervision. At present, there is great variation in the provision of supervision in postgraduate specialties, with specialties such as psychiatry in the UK dedicating large quantities of time to provide their trainees with regular, often weekly, supervision from a senior clinician. This is not replicated in most other postgraduate secondary‐care specialties.

Evaluation of a prospective student's or doctor's level of tolerance of ambiguity might in the future aid high‐stakes selection processes, such as entry to medical school or postgraduate specialties. However, we would express caution in doing so without further consideration of the potential impact on the wider workforce and the supply of trainees for postgraduate specialties.^49^

### 4.4 Future research

To build upon and complement existing research, future studies that are longitudinal in design, multicentre, include medical students and doctors in training or are based on a power calculation are now needed. These might be hypothesis led and test a specific component of the conceptual model proposed in Figure 2. Finally, we recommend that future studies use measurement tools with more careful consideration of their validity for the population studied.

## 5 CONCLUSIONS

There appears to be an association between intolerance of ambiguity and reduced psychological well‐being in medical students and doctors. However, the strength and direction of this relationship is unclear. This is hampered by the small number of studies completed to date, the cross‐sectional nature of studies, the small sample sizes of studies and the wide range of measurement approaches used. This is particularly the case when evaluating levels of tolerance of ambiguity, when subcomponents of previous validated tools are often used and scoring is inconsistent.

Subsequently, the research field to date is patchy and fragmented, rather than programmatic and additive with one study building on the next. Our proposed conceptual model, although based on this limited evidence, does provide researchers with a number of testable hypotheses that could be explored through subsequent research. Our hope is that this can advance this field of research through saving unproductive research efforts. Ultimately, we hope that this will take us closer to designing evidence‐based interventions that might support doctors in coping with their often inherently ambiguous medical work, and may reduce the risk of them developing their own problems with stress, burnout or mental health disorders.

## AUTHOR CONTRIBUTIONS

JH: designed the research, carried out data acquisition, screening, extraction and synthesis, quality appraised the data, drafted and critically revised the manuscript, including revisions, and approved the final version for publication. KM: designed the research, carried out data screening and synthesis, drafted and critically revised the manuscript, provided feedback on revisions and approved the final version for publication. Both authors agree to be accountable for all aspects of the work in ensuring that questions related to the accuracy or integrity of any part of the work are appropriately investigated and resolved.

## CONFLICTS OF INTEREST

None.

## ETHICAL APPROVAL

No human subjects were involved. This was a systematic review.

## Data Availability

The research data supporting this publication are provided within this paper.
