# Supplementary material for: Tolerance of ambiguity and psychological well‐being in medical training: A systematic review
Source: Med Educ. 2019 Dec 22;54(2):125–37. doi: 10.1111/medu.14031 (PMC7003828; doi:10.1111/medu.14031)
Supplement: Supplementary file 1 [file MEDU-54-125-s001.docx]

*Table S1: Search strategy for systematic review*

| PsycINFO |  |  |  |
| --- | --- | --- | --- |
|  |  | and | and |
| or | "MEDICAL STUDENTS"/ | "TOLERANCE FOR AMBIGUITY"/ | "ANXIETY DISORDERS"/ |
|  | "UNDERGRADUATE EDUCATION"/ | UNCERTAINTY/ | exp "MENTAL DISORDERS"/ |
|  | "MEDICAL INTERNSHIP"/ | "RESILIENCE (PSYCHOLOGICAL)"/ | exp "OCCUPATIONAL STRESS"/ |
|  | PHYSICIANS/ |  | (burnout OR "burnt out").ti,ab |
|  | (doctor OR doctors).ti,ab |  | "PSYCHOLOGICAL STRESS"/ |
|  | (training).ti,ab |  |  |
|  | (trainee*).ti,ab |  |  |
|  | ("medical school*").ti,ab |  |  |
|  | ("house officer*").ti,ab |  |  |
|  | (registrar*).ti,ab |  |  |
|  | "MEDICAL RESIDENCY"/ OR "MEDICAL EDUCATION"/ OR "PSYCHIATRIC TRAINING"/ |  |  |
| CINAHL |  |  |  |
|  |  | and | and |
| or | "STUDENTS, MEDICAL"/ | ("ambigu*").ti,ab | exp "ANXIETY DISORDERS"/ |
|  | "STUDENTS, UNDERGRADUATE"/ | UNCERTAINTY/ | exp "AFFECTIVE DISORDERS"/ |
|  | "INTERNS AND RESIDENTS"/ | ("toleran*").ti,ab | exp "MENTAL DISORDERS"/ |
|  | exp PHYSICIANS/ | ("intoleran*").ti,ab | exp "BURNOUT, PROFESSIONAL"/ |
|  | ("doctor*").ti,ab | ("resilience").ti,ab | "STRESS, OCCUPATIONAL"/ |
|  | "SCHOOLS, MEDICAL"/ |  | "STRESS, PSYCHOLOGICAL"/ |
|  | ("house officer*").ti,ab |  |  |
|  | "MEDICAL STAFF, HOSPITAL"/ |  |  |
|  | HOSPITALISTS/ |  |  |
|  | "EDUCATION, MEDICAL"/ |  |  |
| Medline |  |  |  |
|  |  | and | and |
| or | "STUDENTS, MEDICAL"/ | ("ambigu*").ti,ab | ANXIETY/ OR "ANXIETY DISORDERS"/ |
|  | "EDUCATION, MEDICAL"/ | UNCERTAINTY/ | DEPRESSION/ |
|  | "INTERNSHIP AND RESIDENCY"/ | (toleran*).ti,ab | exp "MENTAL DISORDERS"/ |
|  | exp PHYSICIANS/ | (intoleran*).ti,ab | "BURNOUT, PROFESSIONAL"/ |
|  | exp HOSPITALISTS/ | "RESILIENCE, PSYCHOLOGICAL"/ | "COMPASSION FATIGUE"/ |
|  | "SCHOOLS, MEDICAL"/ |  | "STRESS, PSYCHOLOGICAL"/ |
|  | ("house officer*").ti,ab |  |  |

*Table S2: Supplementary searches*

| Supplementary searches:  Following journals were ‘hand searched’ for all articles published online or in paper between 1/1/18 – 24/10/18: |
| --- |
| • BMC Medical Education  • Academic Medicine  • Medical Education  • Medical Education Online  • Academic Pediatrics  • Scandinavian Journal of Primary Health Care  • Academic Emergency Medicine  • Academic Psychiatry  • Annals of Emergency Medicine  • Social Science and Medicine |
| Grey literature searches |
| Following terms searched on Google Scholar (10/4/19) and the first 100 Titles and abstracts identified were reviewed: |
| • “Tolerance ambiguity burnout”  • “Tolerance ambiguity stress”  • “Tolerance ambiguity mental health disorder”  • “Tolerance uncertainty burnout ”  • “Tolerance uncertainty Stress”  • “Tolerance uncertainty mental health disorder” |
| Following websites searched for published / commissioned reports (8/5/19): |
| • https://www.asme.org.uk/ (Association of Medical Education - UK)  • https://www.gmc-uk.org/ (General Medical Council - UK)  • https://www.hee.nhs.uk/ (Health Education England - UK)  • https://www.acgme.org (Accreditation Council for Graduate Medical Education – US)  • https://www.aamc.org (Association of American Medical Colleges – US), following terms searches used: “Ambiguity tolerance”, “ambiguity tolerance mental health”, “uncertainty tolerance”)  • https://www.fsmb.org/ (Federation of State Medical Boards – US)  • https://www.came-acem.ca/ (Canadian Association for Medical Education)  • http://www.royalcollege.ca/rcsite/home-e (Royal College of Physicians and Surgeons of Canada)  • https://amee.org (International Association for Medical Education)  • https://www.mededpublish.org (AMEE MedEd Publish - international) |

*Table S3: Psychometric measures: tolerance of ambiguity or uncertainty*

| Scale name | Outcome measures and subscales | Number items | Psychometric analysis* | Clinically contextualised | Studies |
| --- | --- | --- | --- | --- | --- |
| Physicians’ Reaction to Uncertainty 1990^11^ | Stress from uncertainty  Reluctance to disclose uncertainty | 13 items  9 items | Cronbach’s α = 0.90  Cronbach’s α = 0.75 | Yes  Yes | Iannello et al. (2017)^19^, Bachman et al. (1999)^22^ |
| Physicians’ Reaction to Uncertainty 1995^23^ | Anxiety caused by uncertainty  Concern about bad outcomes  Reluctance to disclose uncertainty to patients  Reluctance to disclose mistakes to physicians | 5 items  3 items  5 items  2 items | Cronbach’s α = 0.85  Cronbach’s α = 0.74  Cronbach’s α = 0.76  Cronbach’s α = 0.75 | Yes  Yes  Yes  Yes | Simpkin et al. (2018)^26^, Takayesu et al. (2014)^27^, Cooke et al. (2013)^24^, Kuhn et al. (2009)^25^ |
| Intolerance of Uncertainty scale (IUS-12)^28^ | Prospective anxiety (fear and anxiety based on future events)  Inhibitory anxiety (uncertainty which inhibits action or experience) | 7 items  5 items | Cronbach’s α = 0.85  Cronbach’s α = 0.85 | No  No | Lally et al. (2014)^20^, Cooke et al. (2013)^24^ |
| Tolerance for Ambiguity scale (Geller)^29^ | Unidimensional measure | 7 items | Cronbach’s α = 0.75 | No | Iannello et al. (2017)^19^, Caulfield et al (2014)^30^ |
| Tolerance for Ambiguity scale (Budner)^31^ | Unidimensional measure | 16 items | Cronbach’s α = 0.49 | No | Lally et al. (2014)^20^, Mangione et al. (2018)^33^ |
| Ambiguous Scenario Task AST-D^35^ | Pleasantness component  Vividness component | 24 items for both components | Cronbach’s α = 0.79  Cronbach’s α = 0.82 | No | Klem et al. (2014)^34^ |

*Taken from the original psychometric validation papers

*Table S4: Psychometric measures: psychological wellbeing*

| Scale name | Outcome measure | Studies |
| --- | --- | --- |
| Maslach Burnout Inventory^36^ | Burnout comprised of emotional exhaustion, depersonalisation, reduced personal accomplishment. | Simpkin et al. (2018)^26^, Torppa et al. (2015)^21^, Takayesu et al. (2014)^27^, Cooke et al. (2013)^24^, Kuhn et al. (2009)^25^ |
| Harvard National Depression Screening Day Scale^40^ | Depression | Simpkin et al. (2018)^26^ |
| Job stress questionnaire (JSQ)^42^ | Stress in workplace | Iannello et al. (2017)^19^ |
| GHQ-12 – General Health Questionnaire 12^44^ | Measure of psychiatric morbidity | Lally et al. (2014)^20^ |
| Perceived Stress Scale. 10 items self-reported stress over last month^43^ | Stress over last month | Caulfield et al. (2014)^30^ |
| Professional Quality of Life (ProQOL) scale^37^ | Quality of life at work | Cooke et al. (2013)^24^ |
| Tedium index^38^ | Burnout | Bachman et al. (1999)^22^ |
| PHQ-9^41^ | Depression | Klem et al. (2014)^34^ |
| Shirom-Melamed Burnout Measur^39^ | Burnout comprised of physical fatigue, cognitive weariness, and emotional exhaustion | Mangione et al. (2018)^33^ |
